# Digital technology use intensity and public safety perception: the moderating roles of victimization experience and safety values

**DOI:** 10.3389/fpsyg.2026.1836160

**Published:** 2026-06-03

**Authors:** Zihao Ding, Zhengyang Li, Yang Luo, Yuhao Wang, Zhongyi Liu

**Affiliations:** 1 School of Public Security Management, People’s Public Security University of China, Beijing, China; 2 Investigation College, People’s Public Security University of China, Beijing, China; 3 School of Public Security, People’s Public Security University of China, Beijing, China

**Keywords:** digital technology use intensity, public safety perception, safety values, social cognitive theory, victimization experience

## Abstract

**Introduction:**

Against the backdrop of deepening digitalization and increasingly differentiated public safety concerns, understanding how digital technology use shapes individuals’ situated sense of security has become critical. Based on social cognitive theory, this study investigates the association between digital technology use intensity and individuals’ public safety perception, operationalized as perceived neighborhood security after dark, with a specific focus on examining the moderating roles of victimization experience and safety values in this process.

**Methods:**

Utilizing large-scale data from the 2023 European Social Survey (ESS), hierarchical regression analysis was conducted on questionnaire data from 33,181 European residents after data cleaning.

**Results:**

The results reveal that: First, digital technology use intensity is significantly positively associated with individuals’ public safety perception(B = 0.048, *p <* 0.001), supporting that digital technology may serve as an informational empowerment and social support tool. Second, victimization experience demonstrates a positive moderating effect (B = 0.078, *p <* 0.001), meaning that for individuals with victimization experience, the positive association between digital technology use intensity and safety perception is stronger. Finally, safety values exhibit a negative moderating effect (B = -0.019, *p <* 0.001), suggesting that for individuals who place greater emphasis on safety, the positive association between digital technology use intensity and safety perception is weaker, revealing the existence of a “safety values paradox.”

**Discussion:**

These findings suggest a modest but robust psychological empowerment association of digital technology use intensity in relation to public safety perception, as examined through the lens of perceived neighborhood security, and uncover the complexity of its relationship with safety perception as well as key boundary conditions. The conclusions provide critical insights for public communication strategies and digital governance aimed at fostering informed and resilient communities in the digital era.

## 1 Introduction

Against the dual backdrop of converging digital technology and social safety risks, global uncertainties and unpredictable risk factors have proliferated significantly. The formation mechanism of public safety perception has become increasingly complex, emerging as a core issue concerning social psychological stability and public wellbeing. According to data from the International Telecommunication Union, the global internet penetration rate has reached 74%, and digital technology has become a core channel for information dissemination and social connection. Meanwhile, data from the 2023 European Social Survey (ESS) employed in this study reveal that among 42,046 respondents across 28 European countries, nearly one in five residents feel unsafe or very unsafe when walking alone in their local area after dark, with this proportion varying significantly across countries and groups. As digital technology increasingly serves as a carrier for risk information, while public perception of everyday safety becomes increasingly differentiated, the formation mechanism of safety perception is no longer a simple matter of technology affecting perception. Instead, it involves a complex psychological process intertwining individual cognition, environmental information, and social support. Thus, exploring the relationship between digital technology use intensity and public safety perception at the micro level has become a critical issue demanding attention in social psychology research in the digital era.

Existing research predominantly operates within the Social Amplification of Risk framework (Kasperson et al., 1988), highlighting the pivotal role of digital technologies, exemplified by the internet, as information media in disseminating and amplifying macro-level risk signals. For instance, Shangguan and Xu (2022) demonstrated that internet use exerts a significant positive effect on public risk perception, with online political participation and trust in government serving as chain mediators. Similarly, Li and Gu (2023) found that differentiated interpersonal trust, including particularized trust in close acquaintances as well as generalized trust in strangers, plays distinct mediating roles in the relationship between internet use and risk perception. The permeation of digital technology into the risk society may trigger such social amplification, thereby exerting a latent influence on citizens’ risk perceptions and emotions (Nakayama et al., 2019). Research in the Latin American context indicates that if the cultivation and construction of individual safety perception fail to reach an adequate level, it can readily precipitate widespread public panic and social instability. Such a scenario not only undermines citizens’ trust in government and their sense of social identity but also weakens societal cohesion and willingness to comply with norms (Corbacho et al., 2015).

However, when shifting the research perspective from macro-level risks to micro-level public safety perception, which is closely tied to individuals’ daily lives, existing studies reveal notable theoretical limitations. Risk perception is a rich and multifaceted concept (Kasperson et al., 1988). While attention has been paid to macro-level risk perception, the safety perception formed by the public’s perceived risks in everyday micro-level contexts constitutes an equally vital dimension concerning social psychology and public wellbeing. Public safety perception specifically refers to an individual’s direct psychological feeling regarding personal and property security within familiar daily spaces such as neighborhoods and streets (Xu and Zhang, 2022; Zou, 2025). Its formation mechanism may differ fundamentally from that of macro-level risk perception directed at abstract systems. Extant research has overly emphasized the “risk amplification” effect of digital technology while generally neglecting its positive impacts on various aspects such as trust in government (Lu et al., 2020), psychological happiness (Hu et al., 2024), self-rated health (Lyu and Sun, 2021), and the quality of life among older adults (Silva et al., 2018). In fact, studies on the influence of digital technology on individual development have revealed that its effects can not only amplify risks but also profoundly shape behaviors, social interactions, cognition, and even mental health (Benvenuti et al., 2023). Digital technology can provide users with precise local public safety updates, security knowledge, and maintain immediate social connections. These functions may positively contribute to an individual’s public safety perception by strengthening their sense of environmental control and perceived social support. For instance, a psychological study focusing on unmarried adults in Indonesia found that in a society where marriage is the norm, the internet serves as a crucial medium for expanding social connections, alleviating loneliness, and enhancing wellbeing among single individuals (Himawan et al., 2022).

Moreover, the psychological correlates of digital technology use are by no means homogeneous; rather, they are profoundly moderated by individual traits and life experiences. On the one hand, victimization experience, as a significant negative life event, can fundamentally alter individuals’ risk perception patterns and information processing strategies (Abbott and McGrath, 2017). It has demonstrated that victimization experience can reshape individuals’ cognitive processing, for instance by triggering rumination, which in turn influences emotional regulation and behavioral responses (Wang et al., 2026). On the other hand, individuals’ intrinsic safety values—that is, their prioritization of the importance of safety—guide their attention allocation, potentially making them more sensitive to online risk information (Huang et al., 2018), a pattern also reflected in the relationship between organizational safety climate and employee safety behaviors (Song et al., 2016). Unfortunately, existing research has not yet systematically incorporated these two crucial individual-level variables into the analytical framework, making it difficult to reveal the specific boundary conditions and underlying mechanisms through which digital technology use intensity is related to individuals’ public safety perception.

In light of this, the present study aims to build on and extend the existing research tradition by shifting the focus from macro-level risks to the domain of micro-level public safety. Specifically, it seeks to investigate the association between digital technology use intensity and individuals’ micro-level public safety perception, operationalized as perceived neighborhood security after dark, as well as the roles played by individuals’ victimization experience and safety values within this process.

## 2 Literature review and hypothesis development

### 2.1 Social cognitive theory

Social Cognitive Theory serves as a core theoretical framework for analyzing the dynamic interplay among individual cognition, behavior, and the environment (Bandura, 1986). The core tenet of this theory posits that an individual’s psychological functioning is the result of continuous, bidirectional interactions among cognitive, behavioral, and environmental factors, rather than being determined by any single element. Among these, self-efficacy, defined as an individual’s belief in their capability to organize and execute the courses of action required to manage prospective situations within a specific domain, constitutes a central motivational factor within the theory. It profoundly influences an individual’s choice of activities, level of effort, and perseverance in the face of difficulties.

In this study, Social Cognitive Theory provides a precise analytical perspective for understanding the complex relationships among digital technology use intensity, individual traits, and public safety perception. Specifically, the behavioral aspect of digital technology use intensity offers individuals rich sources of vicarious experience and verbal persuasion, which are key pathways for enhancing an individual’s self-efficacy regarding safety. Concurrently, digital technology, as an environment, continuously influences an individual’s safety-related cognition through its informational content and social functions. Individual cognitive factors, such as prior victimization experience and the degree of importance placed on safety, will systematically moderate how they interpret the online environment, select specific online behaviors, and ultimately determine the net association of these behaviors and environmental information on their sense of security. Therefore, grounded in Social Cognitive Theory, this study constructs an integrated analytical model aimed at systematically investigating how digital technology use intensity, through its interaction with individual cognitive factors, is related to an individual’s public safety perception.

### 2.2 Digital technology use intensity and individual public safety perception

Public safety perception is typically measured through contextual indicators such as “walking alone at night in one’s residential area”, representing an immediate, situational psychological assessment of personal and property security within an individual’s familiar daily living spaces (Zou, 2025). Unlike macro-level perceptions of overall societal safety, its formation relies not only on external risk information but, more crucially, on the individual’s sense of control over that environment, their familiarity with it, and confidence in their ability to cope. Digital technology use intensity serves as a key indicator measuring the depth of an individual’s integration with digital technologies. Relevant studies have shown that the internet can significantly enhance both the quantity (Jeon and Nasr, 2016) and the precision (Mondria et al., 2010) of information acquisition through technological empowerment, allowing the public convenient access to precise local public safety updates, community crime alerts, and practical security knowledge. This type of highly contextualized, actionable, and localized information constitutes a cognitive resource that reduces environmental uncertainty. It can greatly dispel the “fear of the unknown” caused by information asymmetry, thereby strengthening an individual’s sense of environmental control and perceived neighborhood security based on accurate cognition. Existing research confirms that asymmetry in individual information acquisition can lead to a decrease in trust (Slovic, 1993), predisposing individuals to make negative judgments and consequently lowering their sense of security (Siegrist et al., 2005).

From the perspective of Social Cognitive Theory, digital technology also serves as a powerful tool for enhancing self-efficacy. Individuals who frequently use digital technology to obtain security information and learn coping strategies can continuously accumulate and strengthen their “safety self-efficacy,” their confidence in their own protective abilities and capacity to handle potential threats, through observational learning and knowledge acquisition. The accumulation of this efficacy precisely constitutes the psychological foundation for the formation of public safety perception. More importantly, the online environment fostered by digital technology can serve as an effective channel for social support (Hu et al., 2023). Related research indicates that a sense of security stems from an individual’s subjective perception of the available support (Cohen and Wills, 1985), and that social support contributes to a perception of a secure environment (Zhou et al., 2019). Through instant messaging and social platforms, digital technology enables individuals to maintain close connections with family, friends, and neighbors, establishing a promise of “response-upon-danger” social support. Perceived social support itself, even if not actively mobilized, is a significant psychological resource that can directly help strengthen an individual’s psychological resilience when facing potential threats. This sense of embedded, daily connection can effectively buffer the potential anxiety of isolation and helplessness (Wu et al., 2023), transforming the abstract concept of “social safety” into concrete, reliable interpersonal assurance. Furthermore, emotional resolution following a security incident is crucial for the restoration of safety perception. Frequent users of digital technology can quickly obtain empathetic support within social networks, thereby reducing the traumatic impact of such events (Sippel et al., 2015).

Therefore, this study proposes a dialectical perspective: within the realm of micro-level, concrete public safety perception, the empowering functions of digital technology may outweigh its potential risk amplification function. It can contribute to individuals’ sense of security by transforming them from passive recipients of risk information into proactive agents who actively acquire safety knowledge, connect with social support, and elevate their sense of control and self-efficacy. Based on the above arguments, this study proposes the following research hypothesis:

*H1*: Digital technology use intensity is positively associated with individuals' public safety perception.

### 2.3 Moderating effects of victimization experience

Victimization experience heightens sensitivity to crimes, elevates fear of victimization and risk perception, and leads to a decline in public safety perception (Abbott and McGrath, 2017). It also motivates individuals to compensate for the loss of psychological resources (Hobfoll, 1989) and creates a more urgent need for safety-related information and support (Rogers, 1975). Recent research has further revealed the deep-seated mechanisms through which victimization alters individual cognitive and behavioral patterns. Bartelen et al. (2024) found that childhood poly-victimization significantly affects adulthood aggression by altering impulsivity traits, particularly lack of premeditation, and that male’s exhibit greater vulnerability to the adverse effects of victimization. This finding suggests that victimization not only changes the content of risk perception but also transforms information processing and coping strategies—victims may be more inclined to seek external resources to restore their sense of control.

Digital technology, characterized by vast amounts of information, ease of access, and anonymity in interaction, serves as a crucial channel for individuals with victimization experience to obtain resources. In the context of public safety, individuals with victimization experience are more likely to selectively attend to local crime news, security prevention knowledge, and legal rights information during their digital technology engagement. This directional information acquisition can be categorized into official media and self-media. Browsing official media information contributes to enhancing the subjective sense of safety (Hou, 2022). As “high-risk perceivers,” the digital technology use intensity of individuals with victimization experience may produce a stronger “cultivation effect” (Gerbner et al., 1994), making the role of online security information in shaping their sense of safety more significant.

Meanwhile, victimization is often accompanied by psychological trauma and a sense of social isolation. Social media and online communities on digital platforms provide spaces for individuals to obtain emotional support, share experiences, and receive peer advice (Liu and Brown, 2014). The understanding and assistance gained online through digital technology can buffer the psychological stress caused by victimization and help rebuild a sense of belonging and social trust (Nabi et al., 2013). This suggests that high intensity of digital technology use can bring richer online social support to individuals with victimization experience, amplifying the positive effect of digital technology use intensity on public safety perception.

Based on empirical references from existing literature, this study proposes the following hypothesis:

*H2*: Victimization experience plays a positive moderating role in the relationship between digital technology use intensity and an individual's public safety perception. Specifically, for individuals with victimization experience, the positive predictive association of digital technology use intensity with such safety perception is stronger than for those without victimization experience.

### 2.4 Moderating effects of safety values

Safety values refer to an individual’s value recognition and decision-making criteria for judging safety-related behavioral options, reflecting the priority of safety in their value ranking (Huang et al., 2018). Existing research often regards them as a protective factor promoting safety behaviors (Henriikka et al., 2016). However, for users with significant gaps in digital technology engagement capabilities, in the highly saturated information environment of the internet, high safety values do not simply promote the enhancement of public safety perception.

High safety values guide individuals to form an attentional narrowing toward threat signals, causing cognitive resources to be over-allocated to safety threat-related information and triggering selective attention bias (Kahneman, 1973). In addition to this intra-individual cognitive mechanism, the external information environment on digital media may further exacerbate the attentional narrowing effect among individuals with high safety values. Recent large-scale sentiment analysis of nearly 30 million social media posts from 182 U. S. news sources over a decade (Knutson et al., 2024) revealed that biased news sources (both left- and right-leaning) produce significantly more high-arousal negative (HAN) affective content than balanced sources. Moreover, HAN content is not only more prevalent but also more likely to be reposted, especially by biased sources. This implies that when individuals with high safety values engage with digital technology, they are disproportionately exposed to emotionally charged, threat-related material that is algorithmically amplified due to its high engagement value. Consequently, the already heightened selective attention to threat cues may be further intensified by the sheer volume and virality of HAN content in the online environment.

Digital technology users who primarily use the internet generally exhibit a preference for focusing on negative environmental risk information; they are more willing to capture negative information in the public safety domain rather than positive information (Eriksson and Coultas, 2014). Existing research also indicates that these users pay more attention to negative environmental risk information (Zhang et al., 2019). For individuals with high safety values, greater digital technology use leads them to persistently prioritize negative public safety incidents and crime reports while filtering out positive safety information, thereby weakening public safety perception. Furthermore, when this highly sensitive information processing mode combines with high intensity of digital technology use, individuals are exposed to public safety risk information far exceeding their cognitive load, thus triggering information overload anxiety (Wang, 2021), which is detrimental to the enhancement of safety perception.

In contrast, individuals with lower safety values have fewer constraints on cognitive resources regarding safety risks, enabling them to process online information in a more balanced manner (Fredrickson, 2001). They are less prone to falling into continuous monitoring of risk information and are more susceptible to the influence of mainstream authoritative media information, understanding objective public safety dynamics, thereby enhancing their subjective public safety perception (Hou, 2022). These individuals can also process information obtained through digital technology more rationally, reducing information overload anxiety. Research on environmental safety indicates that the information-derived empowerment from digital technology use needs to be transformed into an actual sense of control and sense of safety through rational processing (Zhang et al., 2019). This rational information processing mode avoids over-taxing cognitive resources on information about public safety risks. It allows individuals to fill their cognitive gap of uncertainty regarding public safety by acquiring more authoritative information, thereby tangibly enhancing their subjective public safety perception Based on the above literature analysis, we posit that a “safety values paradox” likely exists, leading to the hypothesis proposed in this study:

*H3*: Safety values negatively moderate the relationship between digital technology use intensity and public safety perception. That is, the higher an individual’s level of safety values, the weaker the positive association between digital technology use intensity and public safety perception.

### 2.5 This study

**Figure 1:**
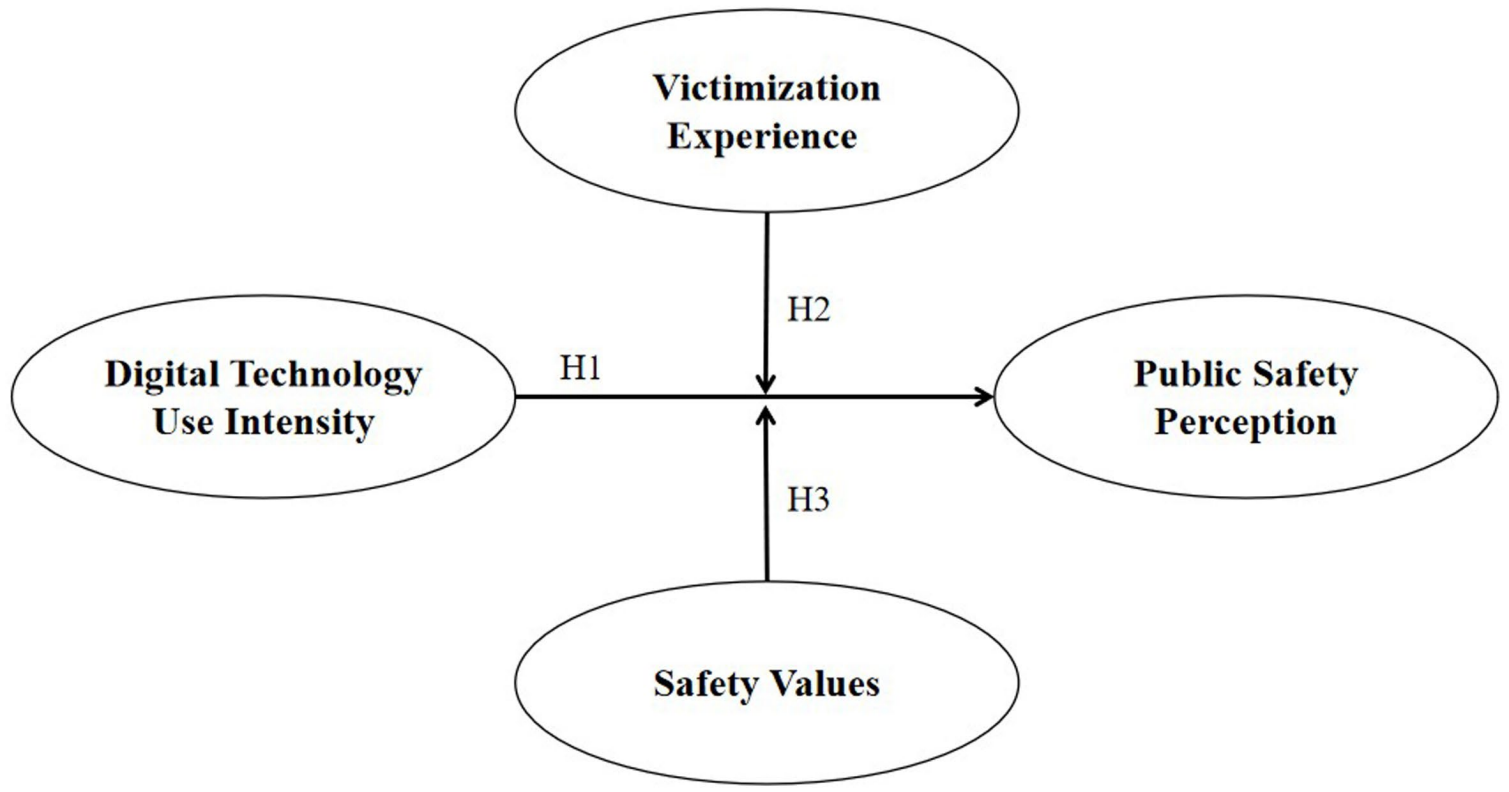
The theoretical model.

This study aims to investigate the association between digital technology use intensity and individuals’ public safety perception, and whether this relationship is moderated by individuals’ victimization experience and safety values. Based on the above theoretical analysis and literature review, this study proposes the following hypotheses: Digital technology use intensity is positively associated with individual public safety perception (Hypothesis 1); victimization experience positively moderates this relationship (Hypothesis 2); safety values negatively moderate this relationship (Hypothesis 3). The theoretical model diagram is shown in Figure 1.

## 3 Methods

### 3.1 Data source

This study utilizes data from the 2023 European Social Survey (ESS). Initiated in 2002 and coordinated by the European Science Foundation, the ESS is a large-scale, cross-national longitudinal research project designed to consistently measure the evolution of social attitudes, values, and behavioral patterns of residents across European countries using rigorous methodologies. Conducted biennially, the survey strictly adheres to probability sampling principles, employing random sampling methods in each participating country. Data are systematically collected through face-to-face interviews, covering multidimensional information such as media use, political participation, social trust, subjective wellbeing, and core values.

The ESS 2023 data are publicly available and were obtained directly from the official ESS website.^1^ After completing a free registration and agreeing to the data use terms, this study downloaded the fully anonymized dataset. The data can be accessed by any researcher for scientific purposes.

The 2023 survey covered the following 28 European countries and regions: Austria, Belgium, Bulgaria, Croatia, Cyprus, Finland, France, Germany, Greece, Hungary, Iceland, Ireland, Israel, Italy, Latvia, Lithuania, Montenegro, The Netherlands, Norway, Poland, Portugal, Serbia, Slovak Republic, Slovenia, Spain, Sweden, Switzerland and the United Kingdom. Among these, the Slovak Republic was excluded from the final analytical sample because the survey item for frequency of social meetings (sclmeet), one of the control variables in this study, was not administered in that country.

The ESS has established unified standards for questionnaire design, translation guidelines, interviewer training, and data validation, ensuring that the collected data are both representative and reliable. Furthermore, all data undergo rigorous cleaning and weighting procedures prior to release, with respondent information anonymized to comply with European data protection regulations and scientific research ethics. Following a strict screening and consolidation process, this study successfully identified 33,181 valid questionnaires, providing a robust data foundation for subsequent analysis.

### 3.2 Variable measurement

#### 3.2.1 Digital technology use intensity

To measure digital technology use intensity, this study selected “Internet use, how often” from the 2023 ESS database as the indicator. The questionnaire asks respondents: “People can use the internet on different devices such as computers, tablets and smartphones. How often do you use the internet on these or any other devices, whether for work or personal use?” Response options include “Never,” “Only occasionally,” “A few times a week,” “Most days,” and “Every day,” corresponding to scores of 1 to 5, respectively. Thus, higher scores indicate greater digital technology use intensity.

#### 3.2.2 Public safety perception

The dependent variable is individual public safety perception. The rate of residents’ nighttime walking serves as an international standard for measuring regional public safety perception. In existing survey research, to confine safety perception within the domain of “public safety,” questions typically ask about residents’ sense of safety in security environments, such as feeling safe when walking alone at night in their residential area (Zou, 2025). This study utilizes the item “Feeling of safety of walking alone in local area after dark” from the 2023 ESS database to measure individual public safety perception. Respondents are asked: “How safe do you—or would you—feel walking alone in this area after dark? Do—or would—you feel...” Based on their feelings, respondents choose from “Very safe,” “Safe,” “Unsafe,” and “Very unsafe.” For the purposes of quantification and regression analysis in this study, the variable was coded with scores of 4, 3, 2, and 1 corresponding to the four options, respectively, where higher scores indicate greater individual public safety perception.

#### 3.2.3 Victimization experience

This variable is measured using the item “Respondent or household member victim of burglary/assault last 5 years” from the 2023 ESS database. In this item, the term “assault” refers to physical assault. The survey question was: “Have you or a member of your household been the victim of a burglary or assault in the last 5 years?” Following data processing in this study, a response of “YES” was coded as 2 points, and a response of “NO” was coded as 1 point.

#### 3.2.4 Safety values

This variable is measured using the item “Important to live in secure and safe surroundings” from the 2023 ESS database. The question assesses how similar respondents feel to people who believe it is important to live in a safe environment and avoid anything that might threaten their safety. The six response options, ranging from “Very much like me,” “Like me,” to “Not like me at all,” correspond to a decreasing level of emphasis on safety. In this study, these responses were assigned scores from 6 to 1 points, respectively.

#### 3.2.5 Control variables

Individual public safety perception is also influenced by demographic factors as well as by socioeconomic conditions, institutional trust, and social capital. Therefore, we include relevant control variables to more precisely analyze the intrinsic relationships among variables, effectively control for potential confounding effects on the research results, and ensure the scientific rigor, reliability, and accuracy of the research conclusions.

This study selected four individual demographic factors, gender, age, years of education, and employment status, as well as four additional theoretically relevant variables, household income, trust in the legal system, trust in the police, and frequency of social interaction, as control variables. For gender, male was coded as 1 and female as 2. Age refers to the respondent’s actual age in 2023. Years of education refers to the duration of schooling completed by the respondent as of 2023. Employment status is a dummy variable, with employed coded as 1 and unemployed coded as 0. Household income was measured by the decile of household total net income, with higher values representing a higher income position relative to the country-specific distribution. Trust in the legal system and trust in the police were assessed, respectively, by items “how much you personally trust the legal system” and “how much you personally trust the police,” both rated on 0–10 scales where 0 means “no trust at all” and 10 means “complete trust.” Frequency of social interaction was measured by the item “how often socially meet with friends, relatives or colleagues,” coded from 1 (never) to 7 (every day).

### 3.3 Statistical analysis

All analyses were conducted using SPSS 26.0. First, common method bias was assessed using Harman’s single-factor test. Subsequently, descriptive statistics and correlation analyses were performed to calculate the means, standard deviations, and Pearson correlation coefficients for all variables, providing an understanding of the basic data structure and preliminary relationships among them. Following this, hierarchical regression analysis was employed to progressively test the direct and moderating effects outlined in the research hypotheses. The variance inflation factor (VIF) was calculated to detect multicollinearity. All VIF values for the main and interaction effects were below 10, indicating no significant multicollinearity issues.

Several robustness checks were conducted to verify the stability of the findings. First, propensity score matching (PSM) was employed to address potential selection bias arising from observable confounders. Second, given the ordinal nature of the dependent variable, all key models were re-estimated using ordered logistic regression, and the relative effect sizes were compared across OLS and ordered logit specifications. Third, to account for unobserved heterogeneity across the 27 European countries in the sample, the full model was re-estimated with country fixed effects by including country dummy variables.

## 4 Results

### 4.1 Common method bias test

This study employed Harman’s single-factor test (Podsakoff et al., 2003) to assess common method bias. The results indicated that a total of five factors had eigenvalues greater than 1, with the variance explained by the first extracted factor being 16.83%, which is below the critical threshold of 40%. This suggests that no serious common method bias exists in the present study.

### 4.2 Descriptive statistics and correlation analysis

Table 1 presents the means, standard deviations, and correlation coefficients for all variables. Significant correlations were observed among the key variables. A significant positive correlation was found between digital technology use intensity and individual public safety perception, providing preliminary support for Hypothesis H1. The independent variable, digital technology use intensity, showed a significant negative correlation with safety values and a significant positive correlation with victimization experience. Victimization experience, safety values, and individual public safety perception were all significantly negatively correlated with each other. The observed correlations within and between the key variable constructs align with expectations, indicating the data are suitable for further analysis.

**Table 1:** Means, standard deviations, and correlation coefficients of each variable.

Variable	Mean ± SD	1	2	3	4	5	6	7	8	9	10	11	12
1	4.27 ± 1.37	1											
2	3.11 ± 0.78	0.129***	1										
3	4.68 ± 1.22	−0.091***	−0.086***	1									
4	0.11 ± 0.32	0.073	−0.078***	−0.043***	1								
5	5.57 ± 2.64	0.104	0.127	−0.047***	−0.020***	1							
6	6.56 ± 2.38	0.029*	0.109	0.028*	−0.030***	0.651	1						
7	1.52 ± 0.50	−0.029***	−0.232***	0.111	−0.018***	−0.023***	0.009**	1					
8	56.73 ± 67.24	−0.145***	−0.033***	−0.001***	−0.021***	−0.018***	−0.015***	0.007**	1				
9	14.30 ± 8.84	0.147	0.028*	−0.083***	0.032*	0.024*	−0.009***	0.001**	−0.001***	1			
10	0.36 ± 0.48	0.259	0.089	−0.031***	0.029*	0.066	0.048*	0.026*	−0.080***	0.070	1		
11	5.62 ± 2.67	0.335	0.132	−0.085***	0.041*	0.125	0.080	−0.091***	−0.087***	0.149	0.418	1	
12	4.83 ± 1.58	0.020*	0	−0.014***	0.020*	−0.005***	−0.003***	−0.014***	−0.01***	0.018*	−0.01***	0.01*	1

1. Digital technology use intensity 2. Public safety perception 3. Safety Values 4. Victimization experience 5. Trust in legal system 6. Trust in police 7. Gender 8. Age 9. Years of full-time education completed 10. Employment status 11. Income level 12. Social meeting frequency. *p < 0.05, **p < 0.01, ***p < 0.001.

### 4.3 Test of the direct effect of digital technology use intensity

This section tests the direct association between digital technology use intensity and individual public safety perception by constructing three hierarchical regression models (see Table 2). As shown, Model M1 includes demographic variables, while Model M2 adds the digital technology use intensity variable on the basis of Model M1. Model M3 further adds four additional control variables: household income, trust in the legal system, trust in the police, and social meeting frequency.

**Table 2:** Direct effects test.

Variable	M1	M2	M3
Constant	3.604*** (233.344)	3.371*** (168.962)	3.114*** (118.326)
Gender	−0.365*** (−44.174)	−0.360*** (−43.698)	−0.351*** (−42.721)
Age	0.000*** (−4.571)	0.000* (−2.157)	0.000 (−1.822)
Years of education	0.002*** (4.177)	0.001 (1.668)	0.000 (0.894)
Employment status	0.148*** (17.147)	0.108*** (12.214)	0.071*** (7.484)
Digital technology use intensity		0.058*** (18.321)	0.048*** (14.682)
Household income			0.015*** (8.324)
Trust in legal system			0.019*** (9.374)
Trust in police			0.019*** (8.558)
Social meeting frequency			−0.002 (−0.858)
R^2^	0.064	0.074	0.089
Adjusted R^2^	0.064	0.073	0.089
F	*F*(4,33,176) = 568.563, *p <* 0.001	*F*(5,33,175) = 526.568, *p <* 0.001	*F*(9,33,171) = 360.144, *p <* 0.001
ΔR^2^		0.010	0.015
ΔF		*F*(1,33,175) = 335.646, *p <* 0.001	*F*(4,33,171) = 141.002, *p <* 0.001

Unstandardized regression coefficients (B) are reported. Values in parentheses are *t*-values. *p < 0.05. **p < 0.01. ***p < 0.001.

The F-test (*F* = 568.563, *p <* 0.001) indicated that years of education (B = 0.002, t = 4.177, *p <* 0.001) and employment status (B = 0.148, t = 17.147, *p <* 0.001) was significantly positively associated with individual public safety perception, while gender (B = −0.365, t = −44.174, *p <* 0.001) was significantly negatively associated with safety perception.

After adding digital technology use intensity to Model 1, the change in *F*-value was significant (*p <* 0.001), indicating that the inclusion of digital technology use intensity contributed explanatory power to the model. Additionally, the R-squared value increased from 0.064 to 0.074, suggesting that digital technology use intensity explained an additional 1% of the variance in individual safety perception. Specifically, the regression coefficient for digital technology use intensity was 0.058 and was statistically significant (t = 18.321, *p <* 0.001), demonstrating that digital technology use intensity was significantly positively associated with individual public safety perception.

Model M3 further incorporates four theoretically relevant controls to address potential omitted variable bias. Among these, household income satisfaction (B = 0.015, t = 8.324, *p <* 0.001), trust in the legal system (B = 0.019, t = 9.374, *p <* 0.001), and trust in the police (B = 0.019, t = 8.558, *p <* 0.001) all exhibited significant positive associations with public safety perception. Social meeting frequency, however, showed no significant association (B = −0.002, t = −0.858, *p* = 0.391).

Importantly, after including these four additional controls, the coefficient of digital technology use intensity remained positive and statistically significant (B = 0.048, t = 14.682, *p <* 0.001), although slightly reduced from B = 0.058 in Model M2. The change in R^2^ from M2 to M3 was ΔR^2^ = 0.015, which was also significant [Δ*F*(4,33,171) = 141.002, *p <* 0.001]. This pattern indicates that while part of the raw association between digital technology use intensity and public safety perception is attributable to socioeconomic and institutional trust factors, a substantial and statistically robust independent association persists.

### 4.4 Test of the moderating effect of victimization experience

This section employs hierarchical regression analysis to examine the moderating effect of victimization experience on the relationship between digital technology use intensity and individual public safety perception (see Table 3). The independent variable (digital technology use intensity) was mean-centered. The moderator variable (victimization experience) was set as a dummy variable. The dependent variable (individual public safety perception) was not processed. The eight control variables—gender, age, years of education, employment status, household income, trust in the legal system, trust in the police, and social meeting frequency—were also left unprocessed.

**Table 3:** Test of the moderating effect of victimization experience.

Variable	M4	M5	M6
Constant	3.114*** (118.326)	3.127*** (119.292)	3.153*** (119.130)
Gender	−0.351*** (−42.721)	−0.353*** (−43.161)	−0.354*** (−43.279)
Age	0.000 (−1.822)	0.000* (−2.005)	0.000* (−1.981)
Years of education	0.000 (0.894)	0.001 (1.236)	0.001 (1.232)
Employment status	0.071*** (7.484)	0.072*** (7.618)	0.072*** (7.659)
Household income	0.015*** (8.324)	0.015*** (8.594)	0.015*** (8.602)
Trust in legal system	0.019*** (9.374)	0.019*** (9.222)	0.019*** (9.189)
Trust in police	0.019*** (8.558)	0.019*** (8.288)	0.019*** (8.231)
Social meeting frequency	−0.002 (−0.858)	−0.001 (−0.557)	−0.001 (−0.512)
Digital technology use intensity	0.048*** (14.682)	0.051*** (15.711)	0.045*** (13.509)
Victimization experience		−0.220*** (−17.105)	−0.242*** (−18.282)
Digital technology use intensity × Victimization experience			0.078*** (6.866)
R^2^	0.089	0.097	0.098
Adjusted R^2^	0.089	0.097	0.098
F	*F*(9, 33,171) = 360.144, *p <* 0.001	*F*(10, 33,170) = 356.238, *p <* 0.001	*F*(11, 33,169) = 328.589, *p <* 0.001
ΔR^2^		0.008	0.001
ΔF		*F*(1, 33,170) = 292.595, *p <* 0.001	*F*(1, 33,169) = 47.142, *p <* 0.001

M4 includes control variables and digital technology use intensity. M5 further includes victimization experience. M6 adds the interaction term (Digital technology use intensity × Victimization experience). t statistics are reported in parentheses. *p < 0.05, **p < 0.01, ***p < 0.001.

M3 included the independent variable (digital technology use intensity) along with the eight control variables (gender, age, years of education, employment status, household income, trust in the legal system, trust in the police, and social meeting frequency). M4 added “victimization experience” to the variables in M3. M5 was built upon M4 by further incorporating the interaction term Digital Technology Use Intensity × Victimization Experience.

As shown in the table above, the independent variable (digital technology use intensity) was significantly associated with public safety perception (t = 14.682, *p <* 0.001). The interaction term between digital technology use intensity and victimization experience was also significant (B = 0.078, t = 6.866, *p <* 0.001). This means that when examining the association between digital technology use intensity and safety perception, the magnitude of the association differs significantly depending on the presence or absence of the moderator (victimization experience).

**Figure 2:**
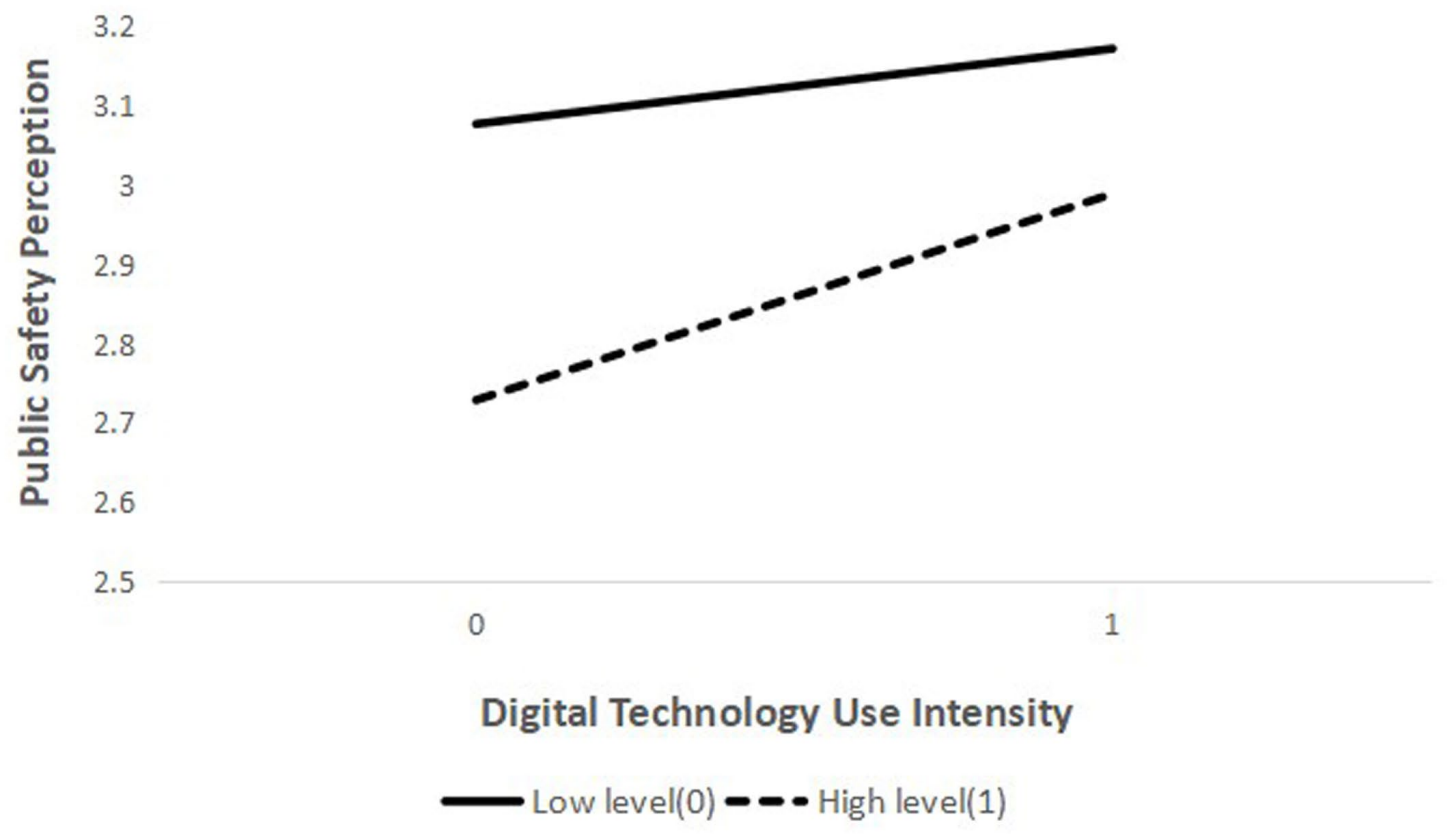
Moderating effect of victimization experience.

To deepen the interpretation of the moderation effect, this study conducted a simple slope analysis. The results are shown in Figure 2. For individuals without victimization experience, the simple slope was B = 0.045, *p <* 0.001, meaning that a one-unit increase in digital technology use intensity is associated with a 0.045-point increase in public safety perception, indicating a modest but statistically significant positive association between digital technology use intensity and public safety perception. For individuals with victimization experience, the simple slope was substantially stronger: B = 0.124, *p <* 0.001. The more than 2.7-fold difference in slope magnitude further indicates that this positive association is more pronounced among individuals with victimization experience.

### 4.5 Test of the moderating effect of safety values

This section employs hierarchical regression analysis to examine the moderating effect of safety values on the relationship between digital technology use intensity and individual public safety perception. The independent variable (digital technology use intensity) and the moderator variable (safety values) were mean-centered. The dependent variable (individual public safety perception) was not processed. The eight control variables—gender, age, years of education, employment status, household income, trust in the legal system, trust in the police, and social meeting frequency—were also left unprocessed. Model M7 included the independent variable “digital technology use intensity” along with the eight control variables. Model M8 added “safety values” to the variables in Model M7. Model M9 was built upon Model M8 by further incorporating the interaction term Digital Technology Use Intensity × Safety values.

As shown in the Table 4, the independent variable (digital technology use intensity) was significantly associated with public safety perception (t = 14.682, *p <* 0.001). Furthermore, the interaction term between the digital technology use intensity and safety values was also significant (B = −0.019, t = −7.102, *p <* 0.001). This result suggests that the association between digital technology use intensity and safety perception differs significantly across varying levels of the moderator (safety values).

**Table 4:** The test of moderating effect of safety values.

Variable	M7	M8	M9
Constant	3.114*** (118.326)	3.258*** (105.559)	3.230*** (103.891)
Gender (ref: male)	−0.351*** (−42.721)	−0.343*** (−41.625)	−0.343*** (−41.561)
Age	0.000 (−1.822)	0.000 (−1.952)	0.000 (−1.852)
Years of education	0.000 (0.894)	0.000 (0.314)	0.000 (0.517)
Employment status	0.071*** (7.484)	0.071*** (7.543)	0.072*** (7.635)
Household income	0.015*** (8.324)	0.014*** (7.969)	0.014*** (7.780)
Trust in legal system	0.019*** (9.374)	0.018*** (8.745)	0.018*** (8.604)
Trust in police	0.019*** (8.558)	0.021*** (9.168)	0.020*** (9.020)
Social meeting frequency	−0.002 (−0.858)	−0.002 (−0.953)	−0.002 (−0.918)
Digital technology use intensity	0.048*** (14.682)	0.046*** (14.178)	0.050*** (15.215)
Safety values		−0.030*** (−8.935)	−0.028*** (−8.297)
Digital technology use intensity × Safety values			−0.019*** (−7.102)
R^2^	0.089	0.091	0.093
Adjusted R^2^	0.089	0.091	0.092
F	*F*(9, 33,171) = 360.144, *p <* 0.001	*F*(10, 33,170) = 332.884, *p <* 0.001	*F*(11, 33,169) = 307.658, *p <* 0.001
ΔR^2^		0.002	0.002
ΔF		*F*(1, 33,170) = 79.831, *p <* 0.001	*F*(1, 33,169) = 50.439, *p <* 0.001

Unstandardized regression coefficients (B) are reported. Values in parentheses are t-values. M7 = Baseline model with all eight control variables and digital technology use intensity; M8 = Model adding safety values; M9 = Full model adding the interaction term (Digital technology use intensity × Safety values). *p < 0.05, **p < 0.01, ***p < 0.001.

**Figure 3:**
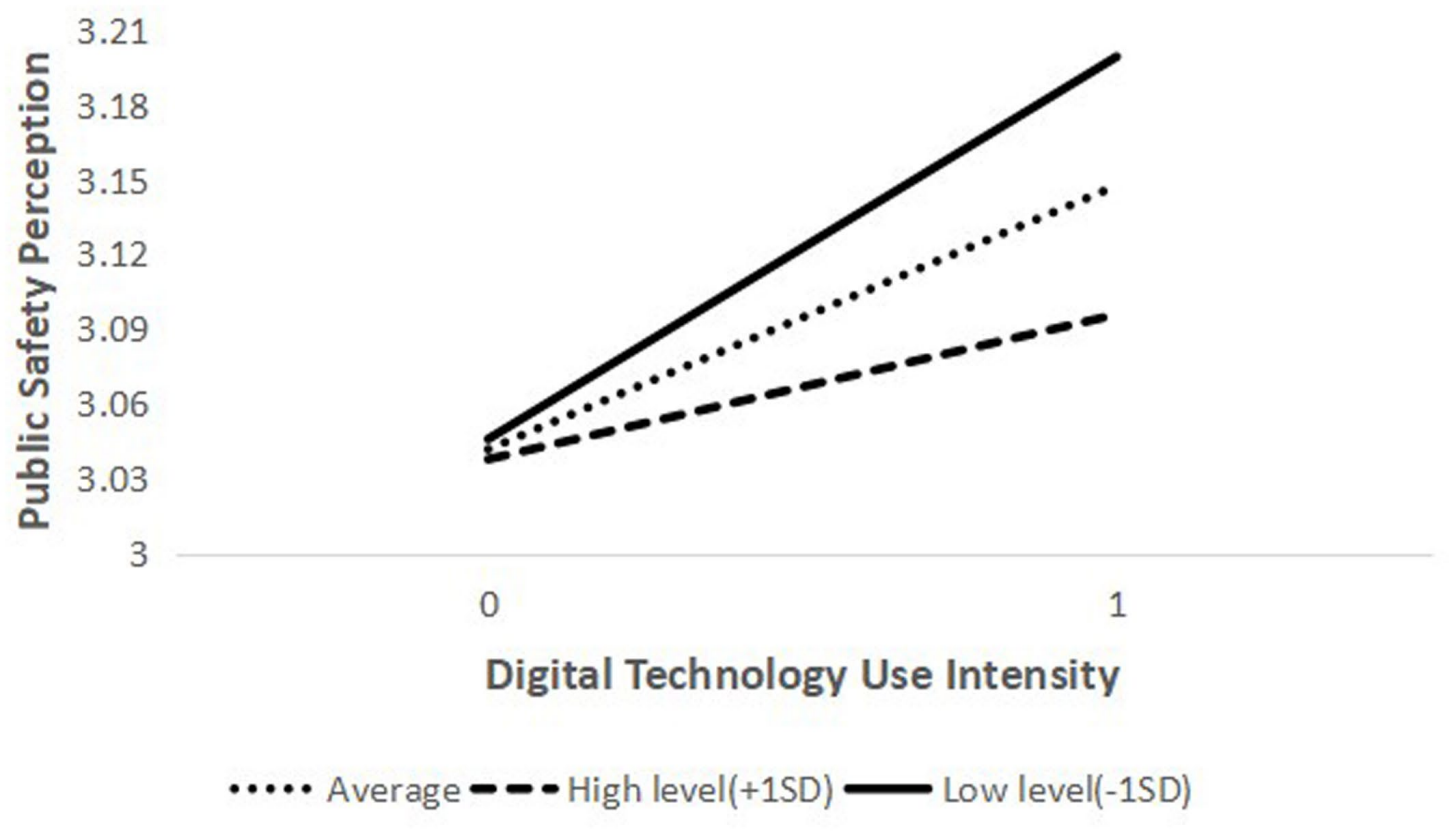
Moderating effect of safety values.

To deepen the substantive interpretation, we conducted a simple slope analysis at three levels of safety values: low (−1 SD; 3.47), mean (4.68), and high (+1 SD; 5.90). The results are presented in Figure 3. At low levels of safety values, the positive association between digital technology use intensity and public safety perception was strongest (B = 0.073, *p <* 0.001), with a one-unit increase in digital technology use intensity associated with a 0.073-point increase in public safety perception. At the mean level, the association was moderate (B = 0.050, *p <* 0.001). At high levels of safety values, the association was substantially attenuated (B = 0.028, *p <* 0.001). The more than 2.5-fold reduction in slope magnitude from low to high safety values provides concrete evidence for the “safety values paradox” identified in our hypothesis testing: the more individuals value safety, the weaker the marginal benefit of digital technology use intensity for their perceived safety.

### 4.6 Robustness check

#### 4.6.1 Propensity score matching (PSM) analysis

To address potential selection bias arising from observable confounders, we conducted a propensity score matching (PSM) analysis. The treatment variable was digital technology use intensity, dichotomized as daily users (netusoft = 5, coded 1) versus non-daily users (netusoft = 1–4, coded 0). The propensity score was estimated using a logistic regression model that included all eight control variables: gender, age, years of education, employment status, household income, trust in the legal system, trust in the police, and frequency of social meetings.

We performed 1:1 nearest-neighbor matching without replacement using a caliper of 0.05 (i.e., 0.05 times the pooled standard deviation of the logit of the propensity score). The final caliper of 0.05 successfully matched 8,842 pairs, yielding a matched sample of 17,684 individuals. This represents 97.8% of the original low-engagement group (9,038 individuals) and 36.6% of the original high-engagement group (24,143 individuals). The high retention rate for the low-engagement group indicates that almost all low-engagement respondents found suitable counterparts among the high-engagement respondents.

Balance after matching was assessed using standardized mean differences (SMDs) for all covariates. Following established guidelines, an SMD absolute value below 0.1 indicates good balance, and values between 0.1 and 0.2 are considered acceptable. As shown in Table 5, before matching several covariates exhibited substantial imbalances (e.g., age: SMD = −0.30; household income: SMD = 0.72; education: SMD = 0.26; employment status: SMD = 0.56). After matching, all SMDs were reduced to below 0.2 in absolute value, with the majority below 0.1. The largest post-matching SMD was for age (−0.17), which remains within the acceptable range. These results confirm that the matching procedure successfully balanced the treatment and control groups on observed confounders.

**Table 5:** Standardized mean differences (SMD) before and after propensity score matching.

Variable	SMD (before)	SMD (after)
Trust in legal system	0.215	0.033
Trust in police	0.083	0.020
Gender	−0.040	0.000
Age	−0.299	−0.169
Years of education	0.255	0.117
Household income	0.715	0.116
Employment status	0.563	0.123
Social meeting frequency	0.025	0.019

SMD (before) calculated from the full sample (*N =* 33,181). SMD (after) calculated from the matched sample (8,842 pairs, total *N =* 17,684). A positive SMD indicates higher mean in the treated group (daily users). Matching was performed using 1:1 nearest-neighbor caliper matching (caliper = 0.05).

To ensure consistent comparability and to adjust for possible residual imbalance in covariates, we re-estimated the regression models on both the full sample and the PSM-matched sample using the binary treatment indicator (daily users = 1, non-daily users = 0) and including the same set of eight control variables. In the full sample, the coefficient of daily digital technology use was 0.176 (*p <* 0.001); in the matched sample, the coefficient was 0.173 (*p <* 0.001). The average treatment effect on the treated (ATT) was 0.184 (SE = 0.0118, t = 15.59, *p <* 0.001, Cohen’s d = 0.24). The full-sample coefficient (0.176), the matched-sample coefficient (0.173), and the unconditional ATT (0.184) are all positive, highly significant, and very close in magnitude, indicating that selection bias due to observable confounders is minimal in this study. Furthermore, the moderating effect of victimization experience remained positive and significant in the matched sample (interaction B = 0.197, *p <* 0.001), and the moderating effect of safety values remained negative and significant (interaction B = −0.041, *p <* 0.001). These results confirm that our findings are robust to selection bias based on observable characteristics.

#### 4.6.2 Ordered logit regression

Given that the dependent variable (public safety perception) is measured on a 4-point ordinal scale, we re-estimated all models using ordered logistic regression. The independent variable, moderating variables, and eight control variables are identical to those in the main OLS analysis.

The results are presented in Table 6. All key coefficients are consistent with the OLS model in terms of sign and statistical significance. Specifically digital technology use intensity is positively associated with public safety perception (ordered logit coefficient = 0.116, *p <* 0.001; OLS B = 0.048, *p <* 0.001). The interaction term between digital technology use intensity and victimization experience is positive and significant (ordered logit coefficient = 0.194, *p <* 0.001; OLS interaction B = 0.078, *p <* 0.001). The interaction term between digital technology use intensity and safety values is negative and significant (ordered logit coefficient = −0.052, *p <* 0.001; OLS interaction B = −0.019, *p <* 0.001).

**Table 6:** Results of ordered logistic regression.

Variable	Main effect model		Victimization moderation model		Safety values moderation model	
	B	SE	B	SE	B	SE
Key variables						
Digital technology use intensity	0.116***	0.008	0.110***	0.009	0.122***	0.008
Victimization experience			−0.572***	0.034		
Safety values					−0.067***	0.009
Digital technology use intensity × Victimization experience			0.194***	0.029		
Digital technology use intensity × Safety values					−0.052***	0.007
Control variables						
Trust in legal system	0.051***	0.005	0.050***	0.005	0.047***	0.005
Trust in police	0.052***	0.006	0.051***	0.006	0.055***	0.006
Gender	0.898***	0.022	0.907***	0.022	0.881***	0.022
Age	0.000*	0.000	0.000*	0.000	0.000	0.000
Years of education	0.001	0.001	0.002	0.001	0.001	0.001
Employment status	−0.181***	0.024	−0.184***	0.024	−0.185***	0.024
Household income	0.041***	0.005	0.043***	0.005	0.039***	0.005
Social meeting frequency	−0.005	0.007	−0.002	0.007	−0.005	0.007
Model statistics						
−2 Log likelihood	70787.482		70530.955		70739.311	
Likelihood ratio *χ*^2^	3100.146***		3373.308***		3224.563***	
df	9		11		11	

*N =* 33,181. B = unstandardized log-odds coefficient; SE = standard error. The dependent variable is public safety perception (1 = very unsafe, 2 = unsafe, 3 = safe, 4 = very safe). The coefficients represent the change in the ordered log-odds of being in a higher category of public safety perception for a one-unit increase in the predictor. All models were estimated using SPSS with the logit link function. *p < 0.05, **p < 0.01, ***p < 0.001.

Furthermore, because OLS coefficients are on a linear probability scale whereas ordered logit coefficients are on a log-odds scale, directly comparing raw coefficient magnitudes is not meaningful. This study therefore compared relative effect sizes, i.e., the ratio of each interaction term coefficient to the main effect coefficient, which are highly consistent across the two models. For victimization experience, the OLS ratio is 1.625 and the ordered logit ratio is 1.672. For safety values, the OLS ratio is −0.396 and the ordered logit ratio is −0.448. This consistency in relative magnitude further supports the robustness of our findings. Taken together, the ordered logistic regression results confirm that our conclusions are not sensitive to model specification.

#### 4.6.3 Country fixed-effects test

Given that the data span 27 countries, we further controlled for unobserved country-level heterogeneity by incorporating country fixed effects (26 dummies, with SI as the reference group) into the model. As shown in Table 7, the coefficient of digital technology use intensity remained positive and significant (B = 0.030, *p <* 0.001), and the interaction effects of victimization experience (B = 0.064, *p <* 0.001) and safety values (B = −0.014, *p <* 0.001) retained their original directions and significance. The country fixed effects were jointly significant (*F* = 124.834, *p <* 0.001), and the model R^2^ increased to 0.182. These results demonstrate that, although systematic baseline differences in safety perception exist across countries, the individual-level associations reported in this study are robust to country-level heterogeneity.

**Table 7:** Robustness check with country fixed effects.

Predictor	(1) Without country FE	(2) With country FE
Digital technology use intensity	0.048*** (0.003)	0.030*** (0.003)
Victimization experience	−0.248*** (0.013)	−0.257*** (0.013)
Safety values	−0.030*** (0.003)	−0.022*** (0.003)
Digital use × Victimization	0.076*** (0.011)	0.064*** (0.011)
Digital use × Safety values	−0.019*** (0.003)	−0.014*** (0.003)
Gender	−0.345*** (0.008)	−0.339*** (0.008)
Age	−0.000* (0.000)	−0.000* (0.000)
Years of education	0.000 (0.000)	0.000 (0.000)
Employment status	0.073*** (0.009)	0.053*** (0.009)
Household income	0.014*** (0.002)	0.020*** (0.002)
Trust in legal system	0.017*** (0.002)	0.012*** (0.002)
Trust in police	0.020*** (0.002)	0.012*** (0.002)
Social meeting frequency	−0.001 (0.003)	−0.001 (0.003)
Country fixed effects	No	Yes (26 dummies)
F-test for country FE		124.834***
Constant	3.276*** (0.031)	3.692*** (0.044)
Observations	33,181	33,181
R^2^	0.102	0.182

Unstandardized regression coefficients (B) with standard errors (SE) in parentheses. Both models include the full set of control variables and two interaction terms, and are estimated within the same GLM framework. The “Without Country FE” model contains all predictors but no country dummies; the “With Country FE” model adds 26 country dummy variables with Slovenia as the reference group. *p < 0.05, **p < 0.01, ***p < 0.001.

## 5 Discussion

Based on Social Cognitive Theory, this study constructed a moderated integrative model to systematically examine the association between digital technology use intensity and individual public safety perception and its boundary conditions. Through analysis of large-scale data from the European Social Survey (ESS), all three research hypotheses proposed in this study were supported. Digital technology use intensity demonstrated a significant positive association with individual public safety perception, and the moderating effects of victimization experience and safety values in this relationship also achieved statistical significance. These findings not only deepen our understanding of the underlying psychological mechanisms through which digital technology use intensity relates to individual public safety perception, but also provide important implications for enhancing public safety perception in the digital era.

Notably, while the three hypothesized effects achieved statistical significance, the observed effect sizes in this study are relatively modest. The full model incorporating demographic control variables, digital technology use intensity, victimization experience, and their interaction term accounted for approximately 8.4% of the total variance in public safety perception (Model M5, R^2^ = 0.084). Similarly, the model examining the moderating role of safety values explained roughly 7.7% of the variance (Model M8, R^2^ = 0.077). Digital technology use intensity uniquely explained an additional 0.5% of the variance in individual safety perception, and the incremental variance attributable to the interaction effects was below 0.5%. Consequently, these results should be interpreted as indicating that digital technology use intensity constitutes a small yet meaningful component within the complex array of factors shaping public safety perception, rather than a primary or decisive determinant. This interpretation underscores that in real-world settings, safety perception remains more substantially contingent upon structural factors such as actual crime rates, neighborhood environmental conditions, and broader socioeconomic contexts.

Although the observed effect size is relatively modest in absolute terms, it still holds academic significance. First, public safety perception is highly shaped by numerous structural factors (e.g., actual crime rates, neighborhood conditions). Detecting a statistically robust association with a single behavioral factor, igital technology use intensity, is non-trivial. Second, the direction of the effect is consistent across all model specifications and robustness checks, indicating that the association is not an artifact of model choice or selection bias. Thus, although the practical implications for individual-level change may be limited, the theoretical contribution, which establishes a reliable empirical link between digital use intensity and perceived safety, remains valuable.

### 5.1 Digital technology use intensity and individual public safety perception

The results show a significant positive association between digital technology use intensity and individual public safety perception, as operationalized through perceived neighborhood security after dark (H1). While the effect size is modest, this finding provides a crucial new perspective for understanding the role of digital technology in the realm of security perception. Dominated by the traditional Social Amplification of Risk framework (Kasperson et al., 1988), prior research has predominantly focused on the amplifying effect of digital technologies on macro-level, abstract risks (Li and Gu, 2023; Shangguan and Xu, 2022). For instance, one study indicated that during the COVID-19 pandemic, the cyberspace derived from digital technologies significantly predicted citizens’ perceptions of health risks (Liu et al., 2021). Li and Wei pointed out that the digital society introduces comprehensive social risks arising from the superimposition of factors such as power and knowledge (Li and Wei, 2020). However, by shifting the focus to micro-level public safety perception, this study demonstrates that in the formation of concrete, situational safety perception, the informational empowerment function of digital technology surpasses its risk amplification effect, emerging as a more prominent factor.

According to Social Cognitive Theory (Bandura, 1986), this positive effect can be understood through two pathways. First, by providing precise local safety updates and security knowledge, more frequent digital technology use could reduce environmental uncertainty and may enhances individuals’ sense of environmental control. This aligns with the core mechanism in self-efficacy theory where control beliefs are built through information and skill acquisition. Second, individuals who use digital technology more intensively may have greater access to online social networks, which offer channels for perceiving social support. This sense of connection might effectively buffer the anxiety associated with isolation and helplessness (Wu et al., 2023), thereby enhancing the public’s safety self-efficacy in coping with potential risks.

The finding that digital technology use intensity positively predicts public safety perception warrants further reflection on the internal tension between competing psychological mechanisms. Although our theoretical framework emphasized the empowering functions of digital technology, it is important to acknowledge that digital technology use may simultaneously operate through risk-amplifying pathways. As Cultivation Theory (Gerbner et al., 1994) and research on the social amplification of risk (Kasperson et al., 1988) suggest, frequent exposure to sensationalized or algorithmically amplified negative content, such as violent crime reports, disaster imagery, or fear-inducing news, could heighten anxiety, amplify perceived vulnerability, and potentially undermine the very sense of security that informational empowerment seeks to bolster.

The positive net association observed in our analysis suggests that, at the population level and within the specific domain of perceived neighborhood safety, the empowering functions of digital engagement may outweigh the risk-amplifying effects. However, the frequency-based measure of digital technology use intensity employed in this study cannot distinguish between instrumental use, such as actively seeking safety information and maintaining supportive social ties, and passive or potentially harmful use, such as algorithmically driven exposure to violent or fear-inducing content. Consequently, the observed positive association reflects the average net pattern across all usage types and does not preclude the existence of heterogeneity in individual experiences. It remains entirely plausible that different types of digital engagement are associated with opposite patterns of perceived safety.

### 5.2 Moderating effect of victimization experience

This study found that victimization experience, on one hand, showed a significant negative association with public safety perception public safety perception (B = −0.220, *p <* 0.001 in Model M5). This aligns with traditional findings in victimology, which posit that victimization creates a novel and unfamiliar sense of vulnerability in victims, heightening their sensitivity to risk and thereby reducing their sense of safety (Perloff, 1983). On the other hand, victimization experience significantly and positively moderated the relationship between digital technology use and safety perception. In other words, for those already at a low point in safety perception due to victimization, the positive association between digital technology use intensity and their sense of safety was stronger compared to non-victims. According to Conservation of Resources Theory (Abbott and Mcgrath, 2017), the acquisition and protection of resources is a primary motivator for individual behavior. When individuals perceive a loss of resources, they experience negative emotions and stress responses. Victims, after suffering harm, often face a dual loss of resources: the physical destruction of a safe environment and the psychological collapse of their sense of control and trust (Wu and Shao, 2015). This state of resource deprivation creates a strong compensatory need, making them more avid seekers of safety-related information (Rogers, 1975). Digital technology precisely provides the tools to meet this need. Individuals can rebuild their cognitive sense of environmental control and enhance their subjective safety by accessing authoritative information from official platforms (Hou, 2022). Simultaneously, they can obtain emotional support through online communities, thereby further amplifying the positive effect of digital technology use intensity on public safety perception. Furthermore, the Planned Risk Information Seeking Model (PRISM) suggests that perceived risk and a sense of informational insufficiency drive instrumental information-seeking behavior (Kahlor, 2010). While non-victims may use digital technology more for recreational purposes, victims, driven by high risk sensitivity and self-protection motives, are more likely to engage in instrumental use of digital technology, such as searching for local crime updates, learning preventive skills, and seeking legal advice. This may help them more effectively translate digital technology use into tangible gains in perceived safety. Related mechanisms are also widely documented in research on health information seeking (So et al., 2019).

### 5.3 Moderating effect of safety values

Hypothesis 3 was supported, as safety values demonstrated a significant negative moderating effect on the relationship between digital technology use intensity and public safety perception. Specifically, the higher the level of safety values, the weaker the positive promoting association of digital technology use on safety perception. This finding supports the existence of a “safety values paradox.”

Individuals with high safety values, who prioritize safety at the apex of their value hierarchy, exhibit a pronounced negative information selective attention bias during digital technology use due to an excessive allocation of cognitive resources toward safety threat information. They are more prone to detecting and remembering negative public safety incidents while filtering out positive safety information (Eriksson and Coultas, 2014). Consequently, even when individuals with high safety values are exposed to positive safety information, its beneficial effect is likely to be attenuated by their prevailing negative emotions, limiting the potential of digital technology use intensity to enhance their sense of safety.

In contrast, individuals with low safety values, whose cognitive resources are not over-occupied by safety threat monitoring, may process online information in a more balanced manner (Fredrickson, 2001). They may be more receptive to positive safety information disseminated by authoritative sources, thereby more readily translating frequent digital technology use into an improved sense of safety. In examining the relationship between social media addiction and depression and anxiety, Lițan (2025) similarly observed that information overload intensifies anxiety symptoms.

### 5.4 Theoretical contributions and practical implications

First, the theoretical contribution of this study lies in expanding the research perspective on digital technology and public safety psychology from macro-level risk perception to micro-level public safety perception, thereby achieving complementary and deepened research at different analytical levels. Second, moving beyond the classic “risk amplification” perspective, this study introduces an integrative theoretical framework incorporating “informational empowerment” and “social support,” providing a new vantage point for comprehensively understanding the dual socio-psychological functions of digital technology. Finally, by empirically testing the moderating roles of “victimization experience” and “safety values,” this study reveals the complexity and boundary conditions of how digital technology use intensity is related to safety perception. Overall, this research investigates the multiple determinants of individual public safety perception, laying a foundation for subsequent empirical studies and practical applications.

Regarding practical implications, this study offers several implications for public communication and digital governance. First, since digital technology use intensity is positively associated with public safety perception, authorities responsible for public communication should recognize digital platforms as empowering arenas for fostering citizens sense of security. Providing accurate, timely, and localized safety information through official digital channels may help reduce environmental uncertainty and contribute to a stronger perceived safety. Second, the significant moderating role of victimization experience highlights the potential value of targeted digital communication strategies for vulnerable populations. For individuals with victimization experience, digital care mechanisms—such as proactively delivering legal aid and psychological support resources through trusted platforms—may amplify the compensatory benefits of digital technology use. Lastly, this study identifies and supports the existence of the “safety values paradox.” For groups with high safety values, information dissemination strategies need optimization to balance risk warnings with the communication of safety achievements. Media literacy education should also be promoted to help them develop rational risk assessment capabilities, thereby breaking the vicious cycle where heightened concern is associated with increased anxiety.

However, this study also has certain limitations. First, this study employs cross-sectional data, which precludes establishing causal-temporal sequences among the variables. Therefore, all reported findings should be interpreted as statistical associations rather than causal effects. To address potential endogeneity concerns, such as omitted variable bias, reverse causality, and selection bias, this study has taken several steps. This study included theoretically relevant control variables in all regression models. This study also conducted propensity score matching (PSM) to balance observed confounders, and re-estimated all models using ordered logistic regression to account for the ordinal nature of the dependent variable. The consistency of results across these alternative specifications increases confidence that the observed associations are not artifacts of model choice or observable selection bias. Nevertheless, because cross-sectional data cannot fully rule out reverse causality or unobserved confounding, future research should employ longitudinal designs (e.g., panel data) or natural experiments (e.g., instrumental variable approaches) to strengthen causal inference and to track how changes in digital technology use intensity co-evolve with public safety perception over time. Additionally, the ESS lacks objective measures of local crime rates or neighborhood safety conditions. We used victimization experience as the closest individual-level proxy for direct exposure to crime and included it as a covariate in moderation models. Future studies should incorporate objective crime statistics to more precisely isolate the net association between digital technology use intensity and public safety perception.

Second, the measurement of digital technology use intensity in the ESS database focuses exclusively on frequency of use and does not distinguish between instrumental use and passive or recreational use. As discussed in the “Discussion” section, although this study finds a positive overall association of digital technology use intensity on perceived public safety, this net association does not preclude heterogeneity across different usage patterns. For instance, heavy passive consumption of negative crime news may reduce safety perception through cultivation effects, whereas active information seeking may contribute to a stronger sense of safety. The single-item frequency measure employed in this study cannot capture this distinction. Future research should adopt multidimensional measures that capture not only frequency of use but also purpose of use, content type, and mode of engagement, in order to disentangle these opposing patterns.

Third, the measurement validity of this study has limitations. The core constructs of this study were all measured using single-item indicators. Although these items have been well-validated in the European Social Survey and enable cross-national comparisons, they cannot fully capture the multidimensional nature of core constructs such as public safety perception and safety values. Specifically, while the item directly taps the importance individuals place on safety, safety values could also include preferences for safety over other competing values (e.g., freedom, convenience, or novelty), situational variations in safety prioritization, or behavioral manifestations of safety concerns. Public safety perception may also include multiple dimensions, such as fear of crime, perceived risk of victimization, and sense of security in different public spaces. Future research should employ validated multi-item scales, such as multidimensional fear of crime scales, to measure these constructs more accurately.

Fourth, although we conducted Harman’s single-factor test to assess common method bias, this test alone has limitations. It is a relatively insensitive diagnostic and cannot fully rule out the possibility that some of the observed relationships are inflated due to shared method variance. Given that all our variables were measured using self-reported items from the same survey, common method bias may still exist. Future research could strengthen causal inference by using multi-method designs (e.g., combining survey data with objective behavioral measures or longitudinal designs) and collecting data from multiple sources (e.g., official crime statistics in addition to self-reports).

Fifth, the effect sizes observed in this study are relatively modest, indicating that digital technology use intensity is only one of many factors influencing public safety perception. Future research should continue to explore determinants of safety perception to develop a more comprehensive understanding of this complex phenomenon.

Finally, while the sample covers multiple European countries, the generalizability of the conclusions to other cultural contexts still requires verification.

## 6 Conclusion

Based on Social Cognitive Theory and an analysis of data from the European Social Survey, this study has elucidated the complex patterns through which digital technology use intensity is associated with individual public safety perception. The findings indicate that digital technology use intensity is significantly positively associated with an individual’s sense of safety by providing localized information and strengthening social support, supporting its psychological empowerment effect at the micro level and demonstrating its capacity to surpass the traditional risk amplification effect. More importantly, victimization experience positively moderates this relationship, highlighting the compensatory role of technology for depleted psychological resources. In contrast, safety values negatively moderate this relationship, consistent with a “safety values paradox” in which high levels of concern are associated with cognitive narrowing and information overload. These conclusions demonstrate that the association between digital technology use intensity and safety perception varies substantially according to an individual’s life experiences and cognitive traits. Therefore, in digital governance, public policy should balance technological inclusivity with targeted interventions. It is essential to both leverage digital technology for broad-based empowerment and design differentiated strategies for specific groups, thereby fostering a more resilient, inclusive, and stable societal mindset toward public safety amidst technological evolution. It is important to emphasize that digital technology use explains only a small fraction of the variance in public safety perception. The formation of safety perception remains fundamentally anchored in objective environmental conditions, institutional performance, and socioeconomic resources. Digital technology should therefore be understood as a complementary tool for public safety governance rather than a substitute for structural improvements in crime prevention and community building.

## Data Availability

The original contributions presented in the study are included in the article/supplementary material, further inquiries can be directed to the corresponding author.

## Footnotes

1
https://www.europeansocialsurvey.org/
